# P-2293. Does Granulocyte-Macrophage Colony-Stimulating Factor as Adjunctive Therapy Affect Mortality in Patients with Hematologic Malignancies?

**DOI:** 10.1093/ofid/ofae631.2446

**Published:** 2025-01-29

**Authors:** Robin Snellings, Sebastian Wurster, Takahiro Matsuo, Ying Jiang, Sung-Yeon Cho, Dimitrios P Kontoyiannis

**Affiliations:** UTH Houston/MD Anderson Cancer Center, Houston, Texas; The University of Texas MD Anderson Cancer Center, Houston, Texas; The University of Texas MD Anderson Cancer Center, Houston, Texas; The University of Texas MD Anderson Cancer Center, Houston, Texas; The University of Texas, MD Anderson Cancer Center, Houston, Texas; The University of Texas MD Anderson Cancer Center, Houston, Texas

## Abstract

**Background:**

Invasive pulmonary aspergillosis (IPA) remains a major cause of illness and death in neutropenic patients (pts) with hematologic malignancies (HM). Because outcomes of IPA are mostly host-driven, adjunct host-targeted immunotherapies such as granulocyte-macrophage colony-stimulating factor (GM-CSF) have been used in cases of refractory or high-risk IPA, though the impact of GM-CSF on outcome remains unclear.

**Figure:**
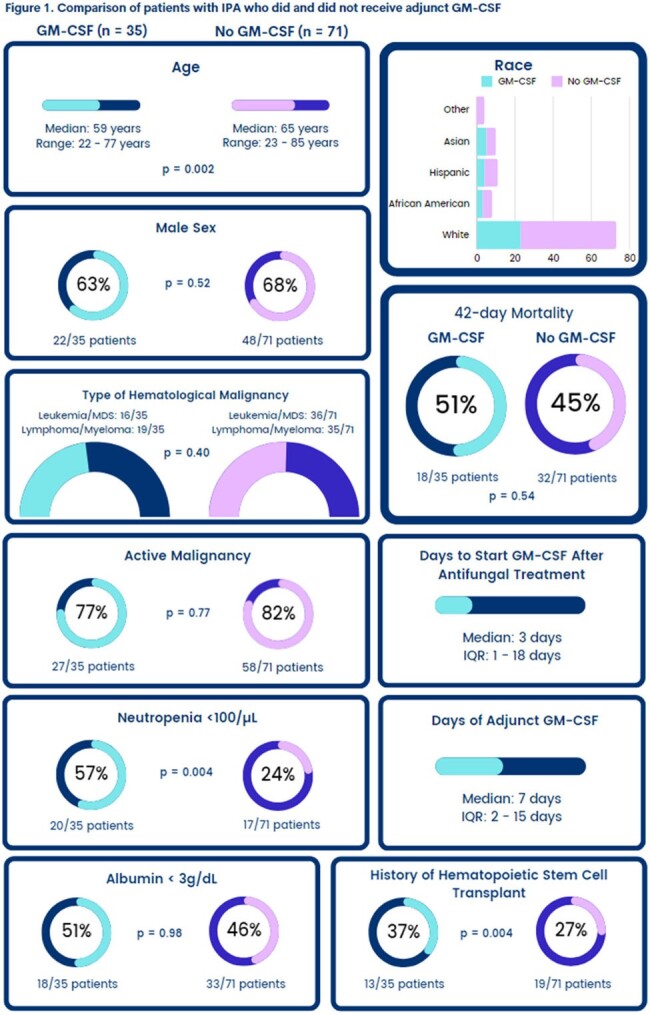

**Methods:**

We reviewed records of all consecutive pts with HM and microbiologically documented IPA from sputum or bronchoalveolar lavage culture at MD Anderson Cancer Center (January 2016 - October 2021). Independent risk factors of 42-day (d) mortality and the impact of GM-CSF on outcomes were analyzed using multivariate Cox regression analysis.

**Figure:**
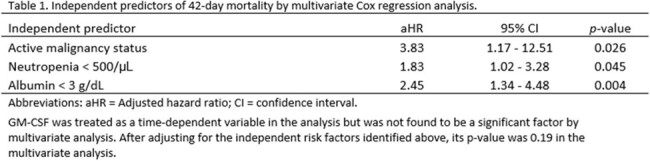

**Results:**

Of the 106 included IPA pts, 52 (49%) had leukemia/myelodysplastic syndrome and 54 (51%) had lymphoma/myeloma; 85 (80%) had active malignancy at IPA diagnosis (Figure 1). Thirty-two pts (30%) had a history of hematopoietic stem cell transplant and 37 (35%) had neutropenia < 100/µL at IPA diagnosis. Thirty-five pts (33%) received adjunct GM-CSF (Figure 1), started at a median of 3 d after initiating antifungal therapy (interquartile range [IQR] 1 – 18). The median duration of adjunct GM-CSF was 7 d (IQR 2 – 15). Forty-two-day all-cause mortality after IPA diagnosis was high (47%) and comparable in pts receiving GM-CSF and those who did not (51% vs. 45%, p = 0.54). Independent predictors of 42-d mortality were active HM (adjusted hazard ratio [aHR] 3.83, p = 0.026), neutropenia < 500/µL (aHR 1.83, p = 0.045), and albumin < 3 g/dL (aHR 2.45, p = 0.004) at IPA diagnosis (Table 1). Considering these co-variables and the timing of GM-CSF, the impact of adjunct GM-CSF on 42-d mortality remained insignificant (p = 0.19) in the multivariate Cox regression model (Table 1).

**Conclusion:**

In a setting of significant IPA burden (i.e., culture-proven disease, neutropenic malnourished pts with active HM) and high overall mortality, adjunct GM-CSF therapy did not affect IPA outcomes. Early culture-independent biomarker-driven IPA diagnosis and immune markers for assessment of the host’s net state of immunosuppression will be pivotal to evaluate whether early preemptive deployment of host-targeted immunomodulators improves IPA outcomes in pts with HM.

**Disclosures:**

Sebastian Wurster, MD, MSc, Astellas Pharma: Grant/Research Support|Gilead Sciences: Grant/Research Support Dimitrios P. Kontoyiannis, MD, AbbVie: Advisor/Consultant|Astellas Pharma: Advisor/Consultant|Astellas Pharma: Grant/Research Support|Astellas Pharma: Honoraria|Cidara Therapeutics: Advisor/Consultant|Gilead Sciences: Advisor/Consultant|Gilead Sciences: Grant/Research Support|Gilead Sciences: Honoraria|Knight: Advisor/Consultant|Merck: Advisor/Consultant|Scynexis: Advisor/Consultant

